# Antenatal diagnosis of chorioamnionitis: A review of the potential role of fetal and placental imaging

**DOI:** 10.1002/pd.6188

**Published:** 2022-06-17

**Authors:** Megan Hall, Jana Hutter, Natalie Suff, Carla Avena Zampieri, Rachel M. Tribe, Andrew Shennan, Mary Rutherford, Lisa Story

**Affiliations:** ^1^ Department of Women and Children's Health St Thomas' Hospital King's College London UK; ^2^ Centre for the Developing Brain St Thomas' Hospital King's College London UK

## Abstract

Chorioamnionitis is present in up to 70% of spontaneous preterm births. It is defined as an acute inflammation of the chorion, with or without involvement of the amnion, and is evidence of a maternal immunological response to infection. A fetal inflammatory response can coexist and is diagnosed on placental histopathology postnatally. Fetal inflammatory response syndrome (FIRS) is associated with poorer fetal and neonatal outcomes. The only antenatal diagnostic test is amniocentesis which carries risks of miscarriage or preterm birth. Imaging of the fetal immune system, in particular the thymus and the spleen, and the placenta may give valuable information antenatally regarding the diagnosis of fetal inflammatory response. While ultrasound is largely limited to structural information, MRI can complement this with functional information that may provide insight into the metabolic activities of the fetal immune system and placenta. This review discusses fetal and placental imaging in pregnancies complicated by chorioamnionitis and their potential future use in achieving non‐invasive antenatal diagnosis.

## Key points

**What's already known about the topic?**

- Chorioamnionitis is present in up to 70% of spontaneous preterm births and is associated with more complications than unaffected spontaneous preterm births
- Diagnosis is postnatal. Antenatal diagnosis by amniocentesis carries a risk of preterm birth, and is not integrated into clinical care

**What does this review add?**

- Ultrasound assessment of the fetal immune system gives structural information and limited functional information, while MRI can complement this with greater functional tissue information
- Imaging may play a role in the non‐invasive antenatal diagnosis of chorioamnionitis

## 1 INTRODUCTION

Chorioamnionitis is acute inflammation of the chorion with or without involvement of the amnion and is evidence of a maternal immunological response to infection. Fetal inflammatory response can coexist and is diagnosed histopathologically when chorionic vasculitis, umbilical phlebitis or funisitis are present.^1^ Detection of a fetal inflammatory response syndrome (FIRS) is associated with poorer outcomes for infants compared to gestation‐matched infants without FIRS.^2^ Overall, chorioamnionitis is implicated in up to 4% of term deliveries, but up to 70% of spontaneous preterm deliveries.^3^ Chorioamnionitis has significant implications for both mother and fetus. In the mother, sepsis can ensue culminating in intensive care admission and maternal death.^4^ Although all fetuses born extremely preterm are at higher risk of mortality,^5^ respiratory, neurological and gastroenterological morbidity, these risks are increased in the presence of chorioamnionits.^6^, ^7^, ^8^ It has been demonstrated that this may, at least in part, be due to the activation of a fetal inflammatory response syndrome (FIRS) that results in marked immune changes that ultimately affect multi‐organ development.

Currently there is no non‐invasive antenatal diagnostic test in clinical practice for chorioamnionitis that is recognised internationally as safe for, and acceptable to, women. Amniocentesis for assessment of the amniotic cavity is the gold standard for intraamniotic infection; it is not routinely used in clinical practice as it is an invasive procedure itself associated with an increased risk of preterm birth (PTB).^9^ Microscopy and gram stain results can be obtained quickly, but culture results take a number of days^10^ whereas clinical decisions often have to be made quickly. Although chorioamnionitis is a mechanism by which preterm labour occurs, expedited iatrogenic delivery is often necessary in an attempt to minimize maternal and fetal morbidity.^11^ The need for expedited delivery is currently determined by non‐specific maternal markers such as abdominal tenderness, fever, purulent discharge and fetal tachycardia, all of which are reflective of late stage infection. However, this is not an effective means of identification of chorioamnionitis^3^ as in nearly one third of cases, chorioamnionitis diagnosed post‐delivery is asymptomatic in the mother.^12^

Whilst in adults systemic inflammatory response syndrome (SIRS) can be diagnosed based on physiological parameters such as temperature and blood pressure, these measures cannot be obtained directly from a fetus in utero to aid with detection of FIRS. Alternative direct biomarkers have been actively sought and there has been interest in interleukin (IL)‐6 measurements as FIRS can be detected postnatally by raised IL‐6 concentrations in the umbilical cord blood, but the practicalities of performing antenatal cordocentesis for a prospective diagnosis are very limited and not without risk.^2^

The focus has shifted to other non‐invasive sampling opportunities for antenatal diagnosis of chorioamnionitis. Previous research has focused on biochemical investigations, including maternal serum, cervicovaginal fluid or amniotic fluid.^13^ Maternal serum markers of infection, such as white cell counts or C‐reactive protein (CRP), have been shown to be ineffective predictors of chorioamnionitis.^14^, ^15^ Multiple cervicovaginal fluid markers have been investigated, some showing moderate specificity and negative prediction, but generally poor positive prediction.^16^ However, their usefulness is likely to be limited: concentrations are known to be transiently raised by cervical cerclage, a common obstetric intervention for women at risk of PTB^17^; and inflammation is a process thought to be associated with most cases of spontaneous preterm labour, so there may be clusters of false positives in women with vascular causes of PTB, such as abruption, which could lead to overtreatment.

Imaging techniques have the potential to provide indicators of chorioamnionitis by assessing components of the fetal immune system, including the spleen, thymus, adrenal gland, liver and the placenta. Both ultrasound and magnetic resonance imaging (MRI) are non‐invasive and have established safety profiles in pregnancy.^18^ Ultrasound can be used to assess the size of fetal organs, as well as blood flow,^19^ and is cheap and readily available. However, image quality can be limited by fetal lie, anhydramnios (often associated with preterm premature rupture of membranes—PPROM—and PTB) and maternal habitus. Although more expensive and in general less widely available (although experience and accessibility are improving), fetal MRI carries significant advantages. MRI uses combinations of static magnetic fields and radiofrequency pulses to align and then alter the magnetisation of protons in tissues to obtain tissue‐specific images. MRI is less limited by fetal lie or anhydramnios than ultrasound, and advanced postprocessing pipelines have been developed to correct for fetal motion improving volumetric analysis of fetal organs.^20^, ^21^ Most importantly, MRI offers functional techniques including T2*, blood‐oxygenation‐level‐dependent (BOLD) MRI, perfusion MRI, and diffusion MRI which can give information on functional tissue properties such as molecular properties and tissue microstructure.^22^, ^23^, ^24^

This review focuses on imaging of the fetal immune system and placenta in the investigation of pregnancies at risk of FIRS and its future potential.

## 2 FETAL IMMUNE SYSTEM: FETAL THYMUS

**FIGURE 1:**
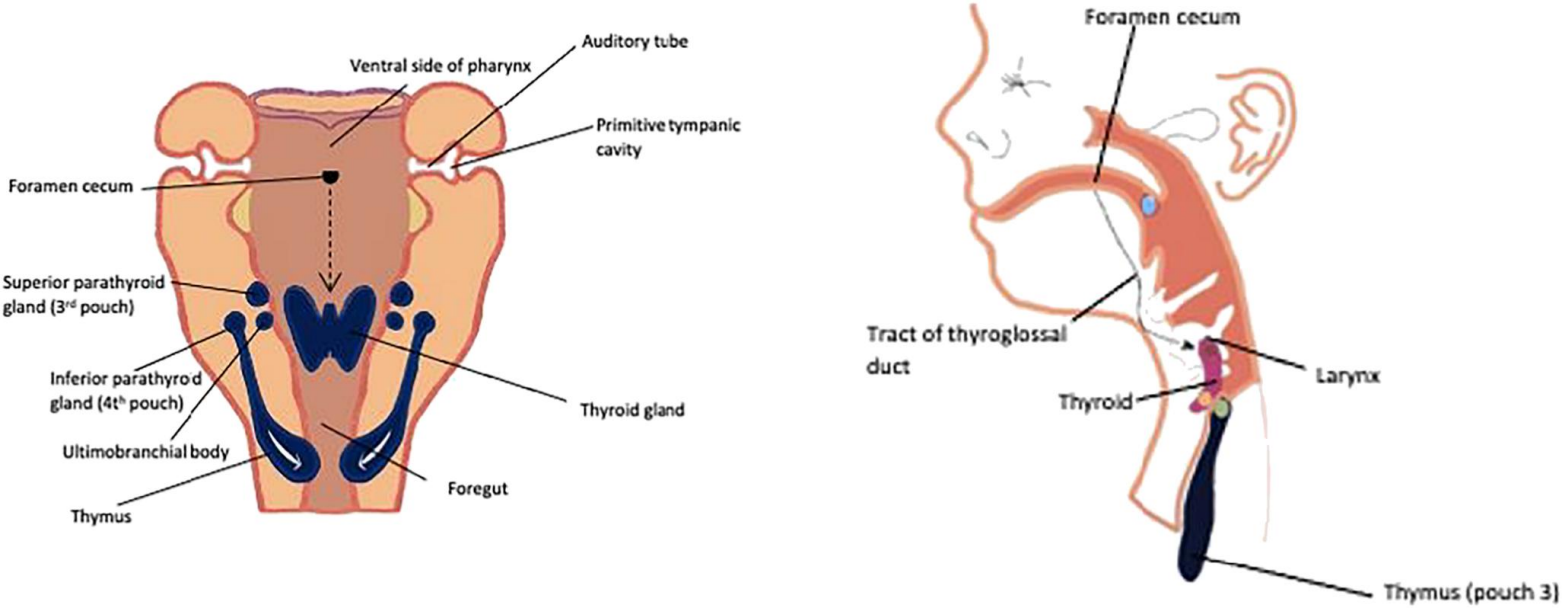
Embryological development of the thymus^25^

The thymus, a bilobular thoracic organ lying in the superior anterior part of the mediastinum and extending towards the neck, is the primary lymphoid organ responsible for T cell development and hence the development of adaptive immunity. Its central role to immunity in the perinatal period renders the thymus a reasonable target of FIRS research. Embryologically, the thymus is derived from the paired third pharyngeal pouch which develop separately into solid masses within the vagal neural crest mesenchyme, descent into the mediastinum only occurs after neck structures are formed and the heart has descended, with the two lobes never fusing, but being united by connective tissue (Figure 1). Functionally, lymphoid stem cell invasion results in 95% of cells being T‐cell lineage by 10 weeks gestation. Macrophages, B cells and erythroblasts are present in smaller quantities. By 17 weeks gestation the thymus is fully differentiated and produces mature thymocytes.^26^ The average mass of the term thymus is thought to be around 10.5 g, with linear growth throughout gestation suspected.^27^ In contrast, the thymus undergoes physiological involution during childhood.^28^

Animal studies have shown that thymic involution can occur in response to chorioamnionitis, which is most likely to be attributable to lymphocyte migration to the blood or inflamed organs, rather than thymic apoptosis.^29^ Studies have also demonstrate that the cortex is more profoundly affected than the medulla.^30^ These changes are not prevented by maternal steroid administration (a common antenatal intervention when PTB is anticipated).^31^ Although this demonstrates proof of principle, it should be noted that animal model chorioamnionitis represents severe infection, and so changes may not be replicated in more mild cases.

In humans, an X‐ray study of 180 neonates has demonstrated a relative reduction in the size of the thymus of infants born to mothers with subclinical chorioamnionitis, compared to age matched controls.^32^ Post mortem studies of fetuses (ranging from late second trimester to term) whose cause of death was linked to extensive chorioamnionitis have similarly demonstrated a reduction in thymic volume, thymic cortical shrinkage, lobule separation, and extensive lymphodepletion.^33^

**FIGURE 2:**
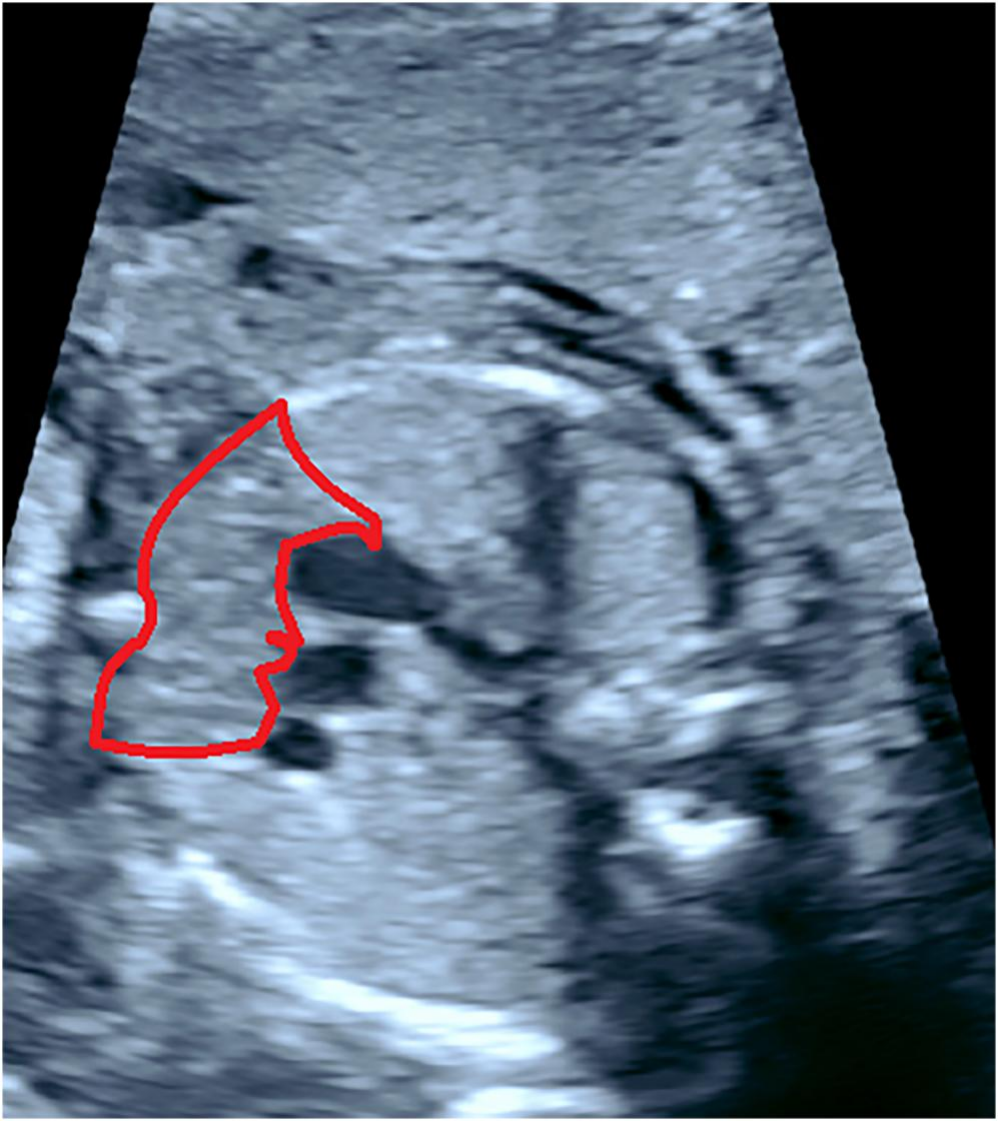
Ultrasound image of the fetal thymus at the level of the three vessel view

Ultrasound identification of the thymus antenatally is utilised in clinical practice as its hypoplasia or absence is associated with congenital diagnoses such as DiGeorge syndrome.^34^ Previous research has demonstrated that thymic volume can be determined via transvaginal ultrasound prior to 18 weeks gestation, and abdominally thereafter, with a study of over 400 patients demonstrating that measurements were obtainable in all cases.^35^ Images are obtained at the three vessel view with the thymus being a well delineated structure anterior to the vessels (Figure 2). The internal mammary arteries define the lateral borders, and Doppler can be used to determine these if there is uncertainty about the border.^36^

Combinations of measurements have been suggested for determining thymic volume, as summarised in Table 1, with measurements below the 5^th^ centile considered evidence of thymic involution.

**TABLE 1:** Methods for ultrasound assessment of the fetal thymus

Measurement	Details of view required
Perimeter^37^	3 vessel view
Transverse diameter^38^	3 vessel view
Thymic area^38^	3 vessel view $thymic\;area=\frac{horizontal\;diameter\;x\;vertical\;diameter\;x\;\pi }{4}$
Thymic:thoracic ratio*^39^	3 vessel view with anteroposteroir diameter of the thymus and anteroposterior intrathoracic mediastinal diameters taken
2D volume*^40^	Maximum transverse, anteroposterior and superioinferior diameters, and maximum transverse areas taken to determine volume
3D volume*^40^	Truncated 3 vessel view with 45° sweep angle. Reconstructed with virtual organ computer analysis system

*Demonstrated in the investigation of congenital thymic dysplasia.

Ultrasound assessment of fetal thymic perimeter and transverse diameter measurements have been demonstrated to predict FIRS and so chorioamnionitis in women with clinically diagnosed PPROM (evidence of pooled amniotic fluid on speculum with or without an adjunct bedside test).^37^, ^38^, ^41^ Thymic area and anteroposterior measurements have shown no demonstrable difference between area in control and PPROM groups, although diameter measurements were significantly reduced in the same study.^38^ Studies commented that sonographers had difficulty obtaining images in cases of severe oligohydramnios or anhydramnios. This is a significant limitation of the technique given the high risk of chorioamnionitis in women with PPROM.^37^, ^41^ Furthermore, the conversion of 2D measurements into approximation of volume is limited by the structure of the thymus which is irregular and varies from fetus to fetus.^42^ As a diagnostic marker of FIRS, fetal thymus measurements outperform conventional biochemistry, with involution shown to occur prior to changes in maternal inflammatory markers, including C‐reactive protein (CRP) and erythrocyte sedimentation rate (ESR).^38^

Meta‐analysis evidence of five studies into thymic involution demonstrated on ultrasound in the antenatal period shows a significant reduction in size of the thymus in fetuses affected by chorioamnionitis. Interestingly this is not upheld in other proinflammatory states such as pre‐eclampsia or intrauterine growth restriction, suggesting that this change is directly related to infection‐related innate immune activation, rather than inflammation itself.^43^ Although this meta‐analysis includes 374 cases, there is high heterogeneity in the sample. There is also no agreement on sensitivity and specificity for clinical practice which would be necessary prior to further clinical implementation, particularly with a view to intervention.

2D MRI has been demonstrated to give comparable transverse diameter and perimeter measurements as compared to ultrasound.^44^ T2 weighted imaging has been shown to give adequate images of the thymus as well as of the total body volume, and normal thymic volumes and ratio to total body volume for gestational ages have been described. Furthermore, evidence of fetal thymic involution in women at high risk of PTB has been demonstrated on MRI, with the majority of patients in the group with involution going on to be diagnosed with histological chorioamnionitis,^45^ implicating MRI as a potentially useful diagnostic tool. One study, directly comparing ultrasound and MRI assessment of the fetal thymus, has demonstrated superior visualization with MRI (97% vs. 80% of cases).^42^

## 3 FETAL SPLEEN

The spleen lies near the gastrointestinal tract, although is embryologically distinct.^46^ A secondary lymphoid organ, it comprises branching arterial vessels which end in a venous sinusoidal system and has roles in filtering erythrocytes, facilitation of iron metabolism and is home to the reticuloendothelial system which is critical for B and T cell (lymphoid cell) immune response.^47^ Exclusively mesodermal in origin, the spleen separates from the pancreas at 13 weeks, with haematopoetic progenitor cells, all of which will become myeloid, functional by 15 weeks gestation.^48^ The fetal spleen is estimated to have a volume of around 6 ml and a length of 36 mm at term, with continuous growth throughout pregnancy (first trimester average length 5 mm, second trimester 28 mm).^49^

Limited animal model studies assessing the fetal spleen in chorioamnionitis exist, and they show variable results. Proinflammatory effects in the spleen have been demonstrated in ovine models of chorioamnionitis induced with lipopolysaccharide,^50^, ^51^ but not when it is induced with IL‐1α.^52^ Rhesus macques monkeys have been shown to develop diffuse reticuloendothelial hyperplasia when chorioamnionitis has been induced through amniotic *Ureaplasma parvum* inoculation.^53^

Conversely, postmortem studies of fetuses and neonates with histological diagnosis of severe chorioamnionitis have demonstrated a severe splenic depletion loss of lymphoid cells with a reduction in T and B cell associated CD cells,^54^ although one study did demonstrate an increase in areas of granulopoesis.^55^ Similarly, postmortem studies of adults who died of sepsis also demonstrated depletion of T and B cells in the spleen.^56^ To our knowledge, no animal or fetal postmortem studies have compared splenic volume depending on the presence of chorioamnionitis.

Ultrasound of the fetal spleen has been demonstrated feasible in the transverse view of the upper abdomen and gestation specific normal ranges established.^57^ Fetal splenomegaly has been described in cases of familial haemophagocytic lymphohistocytosis, although specific details of how measurements were obtained were not provided in the manuscript,^58^ and has been suggested as a screening tool to reduce invasive interventions in suspected fetal anaemia, although the usefulness of this is undetermined.^59^ Details for imaging and suggested normal ranges for fetal circumference have been described, with images obtained from a transverse view of the upper fetal abdomen with the spleen, liver, stomach and left adrenal gland in view; normograms demonstrate essentially linear growth with gestation.^60^ No studies have been identified comparing the transverse diameter of the spleen in patients who go on to be diagnosed with histological chorioamnioinitis with those who do not.

Doppler is widely used in obstetric ultrasound as a proxy marker of altered fetal haemodynamics and so tissue perfusion.^61^ Doppler ultrasound of the fetal splenic vein has also been described: this can be achieved transabdominally in the oblique plane of the fetal abdomen and, like all fetal Dopplers, should be measured in the absence of fetal breathing and fetal movements, and during maternal breathhold if necessary.^61^, ^62^ The normal waveform is a continuous pattern with no change in velocity within the cardiac cycle; pathology is demonstrated by a pulsatile waveform where there is a negative deflection during the cardiac cycle. The mechanism of pulsatile flow is not fully understood, but the authors propose this could be secondary to pulsatile flow from the main portal vein, which is in turn either due to mirroring of atrial contraction through the ductus venosus, or because of pulsations in the hepatic artery. Pulsatile waveform through the splenic vein in women with PPROM has been demonstrated to be significantly associated with histological chorioamnionitis and more so with histological funisitis.^62^ It has also been directly correlated with biochemical diagnosis of FIRS,^63^ with one study demonstrating pulsatile waveform in both the splenic vein and the main portal vein.^64^ The authors hypothesise that this may ultimately be due to FIRS causing cardiac dysfunction and so waveform abnormalities in the precordial veins, although they have not directly demonstrated this pathway. Beyond Doppler findings of uncertain significance, there has been very limited investigation of the spleen in relation to FIRS.

Anatomical variations in the fetal spleen demonstrated on MRI have been commented on in relation to lymphatic malformations and situs anomalies^65^, ^66^; it has also been used as a reference for signal intensity differences in the fetal liver.^67^ Functional properties using T2* weighted imaging have been utilised in fetal animal models and human neonates with an aim to antenatal diagnosis of haemochomatosis.^68^ Given that the changes described are primarily histological rather than associated with changes in mass, the application of diffusion weighted MRI for better understanding of functional properties of the fetal spleen may be merited.

## 4 ADRENAL GLANDS

Proinflammatory cytokines, such as IL‐6, stimulate an acute response in the hypothalamic‐pituitary‐adrenal axis, mainly by stimulating corticotrophin releasing hormone, which via adrenocorticotrophic hormone, will eventually lead to the release of cortisol from the adrenal glands.^69^ Activation of this pathway in the fetus has been indirectly demonstrated via increased amniotic fluid cortisol concentrations in amniotic fluid in a small study of fetuses exposed to intrauterine infection.^70^ The fetal adrenal gland is known to involute shortly after delivery, although there is debate as to whether timing of involution is related to time from delivery or gestational age equivalent.^71^ Histopathological changes in the adrenal gland of stillborn infants with confirmed chorioamnionitis include cortical lipid depletion, luminal haemorrhage and cortical cytolytic degeneration.^72^ In the very low birthweight neonate, there is some evidence of activation of the hypothalamic‐pituitary‐adrenal axis where there is also evidence of thymic involution,^32^ although the effect of maternal steroid administration was not considered. Several studies have noted an enlargement in the fetal adrenal zone in fetuses who go on to be delivered preterm,^73^ although this has not been correlated with presence of chorioamnionitis or FIRS.

Ultrasound assessment of the fetal adrenal gland have been performed^74^ although it is not integrated into routine clinical practice. One study of adrenal 2D ultrasound in low risk women did not demonstrate a significant difference in adrenal size among women who went on to deliver preterm and those who did not; however, there was no differentiation between vascular and cervical causes of preterm birth.^75^ Fetal MRI of the adrenals has been performed: 90%–97% of glands were visible on T2 weighted imaging at 18–30 weeks, although this number falls to 47.5% by 37 weeks.^76^ Given better visibility at earlier gestations, the adrenal glands would be a reasonable site for further investigation in women at high risk of spontaneous preterm birth.

## 5 FETAL LIVER

The liver is responsible for significant fetal haematopoesis from around week 14 of pregnancy.^77^ Ovine studies have demonstrated increased hepatic cytokine production in the presence of chorioamnionitis, as well as antenatal and postnatal abnormalities in lipid metabolism.^78^, ^79^ There is postmortem evidence of increased myelopoesis in cases of chorioamnionitis.^80^ Some ultrasound evidence supports a correlation between increased pulsatility index in the portal Dopplers and intraamniotic infection.^64^ Normal ranges for fetal liver length on ultrasound across gestations have been suggested.^81^ MRI assessment of fetal liver volume has been performed with respect to intrauterine growth restriction.^82^ To our knowledge, no imaging study of the fetal liver with regard to chorioamnionitis has been carried out.

## 6 PLACENTA

Placental histopathology is the current means of diagnosing chorioamnionitis and so a reasonable site for investigation of imaging markers of disease. Histopathologically, chorioamnionitis is subdivided by its maternal and fetal inflammatory response (Table 2).^1^

**TABLE 2:** Histopathological diagnosis of chorioamnionitis^1^

Maternal inflammatory response	
Stage 1: acute subchorionitis or chorionitis	Grade 1: not severe
Stage 2: acute chorioamnionitis – polymorphonuclear leukocytes extend into fibrous chorion and/or amnion	Grade 2: severe – confluent polymorphonuclear leukocytes or subchorionic microabscesses
Stage 3: necrotising chorioamnionitis – karyorrhexis of polymorphonuclear leukocytes, amniocyte necrosis, and/or amnion basement membrane hypereosinophilia	

Fetal inflammatory response	
Stage 1: chorionic vasculitis or umbilical phlebitis	Grade 1: not severe
Stage 2: involvement of the umbilical vein and one or more umbilical arteries	Grade 2: severe – near‐confluent intramural polymorphonuclear leukocytes with attenuation of vascular smooth muscle
Stage 3: necrotising funisitis	

Ultrasound assessment of the placenta is well established in specific obstetric circumstances including: placental location,^83^ abnormal invasion,^84^ and umbilical cord structural pathologies.^85^ In clinical practice, attempts have been made to standardise descriptions of the appearance of the placenta, most commonly via Grannum grading in the late third trimester.^86^ This method has come under scrutiny both for poor correlation with clinical outcome, and high rates of interobserver variability.^87^, ^88^ To our knowledge, it has never been studied in relation to chorioamnionitis. Assessment of placental volume or direct markers of placental function by ultrasound are limited by acoustic clutter and random noise attenuation,^19^ with studied markers of disease like mass, calcification and presence of placental lakes not shown to be clinically useful.^89^ Practically, this results in placental function being monitored with proxy markers such as amniotic fluid volume, evidence of fetal growth restriction, and intermittent or continuous fetal heart rate monitoring. More recent work in the field of pre‐eclampsia and fetal growth restriction, suggests potential value in 3D Power Doppler of the placenta,^90^ but this has not been reproduced in chorioamnionitis.

Placental MRI can be of value anatomically, for example, in characterisation of vascular lesions with T2 weighted images^91^; as well as in providing functional information, such as phenotypical changes in chronic hypertension and pre‐eclampsia,^92^, ^93^ and intrauterine growth restriction.^94^, ^95^, ^96^ More widely, diffusion weighted imaging is already used in assessment of inflammation in inflammatory bowel diseases, and has been shown to have potential in delineating severity of inflammation. A systematic review found that diffusion weighted imaging had a sensitivity of 92% and specificity of 91% for the diagnosis of inflammatory bowel disease, and highlighted the use of apparent diffusion coefficient to stratify severity of inflammation.^97^ Diffusion imaging has been utilised in cardiac myositis^98^ and has been demonstrated to have a diagnostic accuracy of >90% in patients with biopsy proven disease.^99^

More recently, work has been done to investigate the role of placental MRI specifically in relation to chorioamnionitis which demonstrated characteristic changes in the placenta of women who go on to be diagnosed with chorioamnionitis (but were not diagnosed with other placental diseases, such as pre‐eclampsia) as compared to those with low risk pregnancies (Table 3). These changes may be in keeping with microstructural changes described in histopathology, such as increased neutrophils in the chorion and the increase in thrombi in the villous tree.^101^

**TABLE 3:** Observed changes in placental MRI

	Expected change with gestation in normal pregnancy^100^	Changes observed in pregnancy with PPROM and later chorioamnionitis diagnosis^97^	Changes observed in pregnancy with chronic hypertension^92^
Mean T2*	↓	↔/↓^a^	↓↓
Apparent diffusion coefficient (ADC)	↓	↔	↓
Fractional anisotropy (FA)	↑	↑	

^a^ non‐significant trend towards reduction.

Placental ultrasound has a limited remit outside of determining anatomical issues. Functional MRI of the placenta is a relatively new technique although there is evidence supporting its role in evaluation of high risk pregnancies. However, in fetuses at high risk of preterm birth, work is still preliminary and further studies are required to evaluate its role in predicting adverse outcomes. Nonetheless, it is a promising area for investigation.

## 7 CONCLUSION

The diagnosis of chorioamnionitis and resulting FIRS is one that poses a significant clinical conundrum in obstetrics. Currently, obstetricians balance risk and benefits of prolonging gestation against undiagnosed fetal inflammatory response and the risk of maternal sepsis based on poor predictive markers, and so risk unnecessary complications of prematurity or overwhelming fetal, early neonatal or maternal sepsis. Like other obstetric conditions, such as pre‐eclampsia, it is unlikely that a single predictive marker will be sufficient to determine diagnosis, appropriate timing of delivery and likely outcomes.

Imaging markers for the diagnosis of chorioamnionitis and FIRS have considerable clinical potential in influencing management and improving outcomes, and so should continue to be investigated. MRI has the potential to add clarity, particularly regarding functional tissue properties and so should be investigated alongside ultrasound scanning, which is more often used in general obstetrics. The use of multiple metrics, both imaging and biological markers, are most likely to yield useful information and so effort should be put into work that involves modelling for combinations of imaging findings, as well as combinations of imaging and inflammatory marker findings. The combination and temporal relationship between investigations including their ease of use, predicative potential, possible utility for improving neonatal outcomes and cost‐effectiveness need to be considered. Future research into imaging techniques in this field is certainly warranted.

## CONFLICT OF INTEREST

The authors report no conflict of interest.

## Data Availability

Data sharing is not applicable to this article as no new data were created or analyzed in this study.
